# Metabotropic glutamate receptor 3 (mGlu3; mGluR3; GRM3) in schizophrenia: Antibody characterisation and a semi-quantitative western blot study

**DOI:** 10.1016/j.schres.2016.04.015

**Published:** 2016-11

**Authors:** Aintzane García-Bea, Mary A. Walker, Thomas M. Hyde, Joel E. Kleinman, Paul J. Harrison, Tracy A. Lane

**Affiliations:** a Department of Psychiatry, University of Oxford, Oxford, United Kingdom; b Lieber Institute for Brain Development, Baltimore, USA; c Department of Neurology, Johns Hopkins School of Medicine, Baltimore, USA; d Department of Psychiatry & Behavioral Sciences, Johns Hopkins School of Medicine, Baltimore, USA; e Oxford Health NHS Foundation Trust, Warneford Hospital, Oxford, United Kingdom

**Keywords:** Group II metabotropic glutamate receptor, Methods, Human brain, Psychosis, Antibody validation

## Abstract

**Background:**

Metabotropic glutamate receptor 3 (mGlu3, mGluR3), encoded by GRM3, is a risk gene for schizophrenia and a therapeutic target. It is unclear whether expression of the receptor is altered in the disorder or related to GRM3 risk genotype. Antibodies used to date to assess mGlu3 in schizophrenia have not been well validated.

**Objective:**

To characterise six commercially available anti-mGlu3 antibodies for use in human brain, and then conduct a semi-quantitative study of mGlu3 immunoreactivity in schizophrenia.

**Methods:**

Antibodies tested using Grm3^−/−^ and Grm2^−/−^/3^−/−^ mice and transfected HEK293T/17 cells. Western blotting on membrane protein isolated from superior temporal cortex of 70 patients with schizophrenia and 87 healthy comparison subjects, genotyped for GRM3 SNP rs10234440.

**Results:**

One (out of six) anti-mGlu3 antibodies was fully validated, a C-terminal antibody which detected monomeric (~ 100 kDa) and dimeric (~ 200 kDa) mGlu3. A second, N-terminal, antibody detected the 200 kDa band but also produced non-specific bands. Using the C-terminal antibody for western blotting in human brain, mGlu3 immunoreactivity was found to decline with age, and was affected by pH and post mortem interval. There were no differences in monomeric or dimeric mGlu3 immunoreactivity in schizophrenia or in relation to GRM3 genotype. The antibody was not suitable for immunohistochemistry.

**Interpretation:**

These data highlight the value of knockout mouse tissue for antibody validation, and the need for careful antibody characterisation. The schizophrenia data show that involvement of GRM3 in the disorder and its genetic risk architecture is not reflected in total membrane mGlu3 immunoreactivity in superior temporal cortex.

## 1 Introduction

Group II metabotropic glutamate receptors comprise mGlu2 and mGlu3, encoded by GRM2 and GRM3 respectively. They are G protein-coupled receptors, serving primarily as presynaptic autoreceptors, involved in many facets of synaptic plasticity and brain function (Niswender and Conn, 2010). These receptors are implicated in schizophrenia as part of the broader glutamatergic hypotheses of the disorder, in part driven by pharmacological studies showing that group II mGlu agonists can ameliorate deficits caused by NMDA receptor antagonism (Moghaddam and Adams, 1998; for review see Moreno et al., 2009, Moghaddam and Javitt, 2012). This work fostered development of mGlu2/3 agonists as potential anti-schizophrenia treatments, with a high-profile positive clinical trial for one such drug, pomaglumetad methionil (Patil et al., 2007). Though this finding was not replicated, interest in group II mGluRs in schizophrenia and as antipsychotic drug targets has persisted (Lyon et al., 2011b, Fell et al., 2012, Vinson and Conn, 2012, Lane et al., 2013, Ellaithy et al., 2015, De Filippis et al., 2015, Pritchett et al., 2015, Walker and Conn, 2015), and a recent secondary analysis of the clinical trials suggests that pomaglumetad methionil may have antipsychotic efficacy early in the disease and in patients previously exposed to D2 dopamine antagonists (Kinon et al., 2015).

The pathophysiological role and therapeutic potential of group II mGluRs in schizophrenia is complemented by increasing evidence that GRM3 is a risk gene for the disorder (Harrison et al., 2008). Initially reported in candidate gene studies (Egan et al., 2004), the evidence is now markedly enhanced by the finding that the GRM3 locus is genome-wide significant for schizophrenia (Schizophrenia Working Group of the Psychiatric Genomics Consortium, 2014). Notably, the signal is intragenic, supporting the interpretation that the genetic association is to the gene itself, and that it may operate by altering GRM3 regulation and expression (Sartorius et al., 2008, Kleinman et al., 2011, Harrison, 2015).

As part of the characterisation of GRM3/mGlu3 in schizophrenia, several studies have measured expression of the gene in brain tissue (see Harrison et al., 2008, Hu et al., 2015). Studies of GRM3 mRNA do not show clear differences between schizophrenia cases and controls (Ohnuma et al., 1998, Richardson-Burns et al., 2000, Egan et al., 2004, Bullock et al., 2008, Gonzalez-Maeso et al., 2008, Kim et al., 2012) although there may be a modest increase in prefrontal cortex (Sartorius et al., 2008). The latter authors also reported that a GRM3 risk SNP was associated with decreased expression of a transcript isoform which lacked exon 4 and predicted to encode an mGlu3 variant with a novel C-terminus (Sartorius et al., 2006, Sartorius et al., 2008). With regard to studies of mGlu3 immunoreactivity, the data are more variable (see Table 1 for summary of existing studies). A major consideration is that most studies have used antibodies which cross react with mGlu2, or antibodies which have not been well characterised to demonstrate their specificity for mGlu3. The one exception is the antibody generated by Corti et al. (2007), which in Grm3^−/−^ mice showed selectivity. With this antibody, they then demonstrated a reduction of mGlu3 dimer in prefrontal cortex in schizophrenia.

Given the renewed, genomically-driven, focus on GRM3, the question of mGlu3 expression in schizophrenia, and its potential modulation by schizophrenia risk genotype, requires a clearer answer than the prior studies permit. Firstly, by using antibodies that have been sufficiently well characterised. To this end, we have tested a range of anti-mGlu3 antibodies, using brain tissue from Grm3^−/−^ mice, and from Grm2^−/−^/3^−/−^ mice, complemented by transfection of cells with human GRM3 cDNA. We also tested a novel antibody directed at the previously-reported novel mGlu3 variant (mGlu3Δ4; Sartorius et al., 2008). Having characterised the antibodies, we used a validated C-terminal anti-mGlu3 antibody for a semi-quantitative immunoblot study of membrane protein from superior temporal cortex from a series of over 150 patients with schizophrenia and comparison subjects, who were genotyped for a GRM3 risk single nucleotide polymorphism (SNP). The superior temporal cortex is implicated in schizophrenia in terms of alterations in volume (Sun et al., 2009), connectivity (Lee et al., 2009), cytoarchitecture (Eastwood and Harrison, 2003, Beasley et al., 2009), and gene expression (Burnet et al., 1996, Eastwood and Harrison, 2005, Schmitt et al., 2011).

## 2 Materials and methods

### 2.1 Mouse brain tissue

Brains were taken from adult Grm3^−/−^ knockout mice, Grm2^−/−^/3^−/−^ double knockout mice, and wild-type mice (Lyon et al., 2008, Lyon et al., 2011a, De Filippis et al., 2015), snap frozen, and stored at − 80 °C. For protein isolation, a small piece of frontal tissue was cut with a clean razor blade, weighed, and homogenised using membrane extraction buffer and a Dounce homogeniser on ice (*n* = 3 adult female mice per genotype).

### 2.2 Human brain tissue

The demographic details of the human brains used for the main quantitative study are summarised in Table 2 (‘Full series’; for explanation of the ‘Matched series’ see Section 2.9).

The brains were collected at the National Institute for Mental Health (NIMH) and are from a series described and used in previous studies (e.g. Lipska et al., 2006, Eastwood et al., 2010). Briefly, the tissue was obtained with informed consent from the legal next of kin under NIMH protocol no. 90-M-0142. Diagnoses were made by independent reviews of clinical records by two board-certified psychiatrists using DSM-IV criteria. Control subjects were designated as such based on a standardized screening interview with next of kin, in addition to a review of all available medical records and investigators at the medical examiners' offices. Brains were examined macroscopically and microscopically by a board-certified neuropathologist, and all subjects with significant pathological features were excluded (Lipska et al., 2006). Toxicological analysis was conducted for each case using blood and/or brain specimens for drugs of abuse and medications. Control cases with ethanol levels above 0.05 g/dL, or positive for any illicit drugs, or medications above therapeutic levels, were excluded. For this study, a block of superior temporal gyrus (Brodmann area 22) was dissected from a frozen coronal slab of each brain, with white matter trimmed using a dental drill. The block was maintained at − 80 °C until protein extraction, performed as described for mouse brain tissue.

In addition, we used frozen tissue blocks or sections from several ‘test’ brains provided by the Stanley Medical Research Institute for some pilot studies during validation of the antibodies, and for mGlu3 detection in brain regions other than the superior temporal cortex.

### 2.3 Genotyping

Genotyping of rs10234440 (T/C) was performed on DNA extracted from the superior temporal cortex tissue, using a Taqman assay (ID C_341072_10) and a 7900HT real-time PCR system. This SNP lies within the GRM3 region showing genome-wide association to schizophrenia (Schizophrenia Working Group of the Psychiatric Genomics Consortium, 2014) and is also in linkage disequilibrium with another SNP, rs2228595, previously shown to have an effect on expression of a GRM3 mRNA isoform (GRMΔ3Δ4) though not on full-length GRM3 mRNA (Sartorius et al., 2008).

### 2.4 Cell culture and transient transfection

The human embryonic kidney cell line HEK293T/17 (ATCC CRL-11268) was maintained in Dulbbeco's modified Eagle's medium (DMEM; Sigma D6545) containing 4.5 g/L glucose and supplemented with 10% fetal bovine serum (FBS) (Sigma F9665) and 4 mM l-glutamine (Sigma G7513). Cells were seeded at a density of 5 × 10^4^ cells/cm^2^ in Chamber Slides (Nunc™ Lab-Tek™ II Chamber Slide™ System) for immunocytochemistry or T-75 flasks for western blots. Cells were grown for 24 h and then transfected with a PCI neo construct containing the open reading frame for either full length human GRM3 or GRM3Δ4 using a standard polyethylenimine (Aldrich 40,872-7 average MW 25000) protocol. Briefly, human GRM3 construct (533.33 ng/μL; 200 ng/cm^2^) was mixed with 20% glucose at a ratio of 1:3 glucose to DNA, PEI (5.6 mg/mL) was then added to the mix at a ratio of 1:3.3 (PEI to DNA glucose) and incubated for 5 min at room temperature. The mixture was added to fresh culture media (DMEM 4.5 g/L glucose, 10% FBS and 2 mM l-glutamine) and cells were incubated for 24 h, following which, the media was exchanged. Cells were incubated for a further 24 h before harvesting.

### 2.5 Antibodies

We tested several commercially available anti-mGlu3 antibodies (Table 3). The Table also lists the other antibodies used.

In addition, a polyclonal antibody was generated (Cambridge Research Biochemicals, Cambridge, UK) to the novel C-terminus of mGlu3Δ4. The antibody was raised against the peptide sequence TQGSHHPVTPEEC which corresponds to amino acids 465–478 of the variant protein. Crude antiserum was purified by affinity chromatography and used for western blot at 1:500. For some experiments, the peptide was used as a blocking peptide at 0.03 mg/mL (> 50 × the concentration of the antibody).

### 2.6 Western blotting

Total membrane protein from cells, mouse and human brain samples was extracted using a kit (Biovision Incorporated, Milipitas, California, USA), according to manufacturer's instructions, with minor modifications (viz. addition of 100 μM iodoacetamide during homogenisation to prevent receptor aggregation). As described by the manufacturer, the protocol results in two fractions: a ‘total cellular membrane protein fraction’ and a ‘cytosolic fraction’; the former includes the plasma membrane and all organelle and other intracellular membranes, and hereafter is referred to simply as the ‘membrane fraction’.

Total protein concentration was determined using the Bradford assay; membrane protein was diluted to 1 mg/mL and stored at − 20 °C until use. Protein samples were combined with Laemmli loading buffer containing 100 mM DTT and heated at 55 °C for 5 min; this protocol reduced aggregation compared to boiling with β-mercaptoethanol. Protein (10 μg for brain tissue, 1 μg for cells) was loaded in a 4–20% mini-Protean polyacrylamide gel (Bio-Rad 4561095) and run in SDS/Tris/glycine buffer (25 mM Tris-HCl, 250 mM glycine, 0.1% SDS) at 100 V for two hours. Proteins were then transferred to a PVDF membrane (Millipore) in Tris/glycine/methanol buffer (25 mM Tris-HCl, 192 mM glycine, 20% methanol) at 25 V overnight. The purity of the resulting membrane fraction was assessed using anti-N-cadherin and anti-GAPDH antibodies (Table 3), at 1:10,000 and 1:5000 respectively.

PVDF membranes were blocked for 40 min at room temperature with 5% skimmed milk diluted in phosphate-buffered saline with 0.1% Tween (PBST). The primary and secondary antibody incubations were performed at room temperature in PBST with 2% skimmed milk, for 1 h and 40 min respectively. Wash steps before and after addition of secondary antibody consisted of three 10 min incubations in PBST, with a final 5 min wash in PBS prior to addition of Enhanced Chemiluminescence (ECL) reagent (GE Healthcare, Fisher Scientific, Loughborough, UK) as per manufacturer's instructions. The blots were exposed to film (GE Healthcare) and digitally captured using an AlphaImager3400 system.

### 2.7 Immunocytochemistry and immunohistochemistry

The ab166608 antibody was tested for use in immunocytochemistry (in the HEK293T/17 cells) and for immunohistochemistry (in brain sections).

HEK293T/17 cells grown in chamber slides were fixed in paraformaldehyde (4% w/v in PBS) for 15 min at room temperature, washed three times for 5 min in PBS, blocked and permeabilized for 40 min with PBS containing 10% goat serum and 0.1% Triton-100X at room temperature. The primary antibody incubation was performed for 1 h at room temperature. Cells were then washed 3 times for 15 min in PBS and incubated with secondary antibody (Alexa 568; Table 3) for 1 h at room temperature, washed for 3 × 15 min in PBS, then 2 min in distilled water, air dried and mounted in Vectashield Antifade Mounting Medium with DAPI (Vector, H-1200).

Frozen 14 μm sections of mouse brain (*n* = 2–3 mice of each genotype) were fixed in paraformaldehyde (4% w/v in PBS) for 15 min at room temperature, and washed three times for 5 min in PBS. To reduce autofluorescence, sections were incubated for 15 min in 50 mM glycine, and washed three times for 5 min in PBS. Sections were blocked and permeabilized in PBS containing 10% goat serum and 0.3% Triton-100X for 1 h at room temperature. Following an overnight incubation with the primary antibody at 4 °C, sections were washed 3 times for 15 min in PBS and incubated with secondary antibody (Alexa 568; Table 3) for 1 h at room temperature. Finally sections were washed three times for 15 min in PBS, then 2 min in distilled water, air dried and mounted in Vectashield Antifade Mounting Medium with DAPI (Vector, H-1200). As a control, the ab166608 blocking peptide was diluted in blocking solution, at a concentration ten times greater than the antibody, and co-incubated for two hours at room temperature with constant agitation.

### 2.8 Study of mGlu3 immunoreactivity in schizophrenia

For the study of mGlu3 in schizophrenia, the ab166608 antibody was used for western blotting, at 1:50,000. Quadruplicate samples of membrane protein were used for each subject, run as two sets of duplicates on consecutive days. To control for variability between days and gels, two lanes of every gel contained an ‘internal’ protein standard made from a pool of six individuals selected randomly from the brain series; the demographic details of these subjects are shown in Supplementary Table 1. The use of an internal standard has been advocated (McCullumsmith and Meador-Woodruff, 2011) and used in prior studies in the field (Crook et al., 2002, Corti et al., 2007). Beta-actin (1:2,000,000) was measured as a control for protein loading; the abundance of β-actin is reported to be unchanged in schizophrenia (Bauer et al., 2009). The study was conducted and analysed blind to diagnosis, genotype, and other demographic information.

Measurement of the bands was carried out using Image Studio Lite software (LI-COR Biosciences, Cambridge, UK). Two film exposure times were used, to optimise measurement of monomer and dimer bands, as these had different staining intensities, and to avoid film saturation. A box was drawn around each band and the total intensity (the sum of the individual pixel intensities) was determined. The mean value from the quadruplicate readings was used for the analysis. Results for mGlu3 bands were normalised to the internal standards run on that gel; the value for the standard was set to 100; the data presented here are thus arbitrary units, relative to this value.

### 2.9 Statistical analysis

mGlu3 immunoreactivity was inspected for correlations with continuous variables (e.g. age, pH, post mortem interval [PMI]) using the Pearson's coefficient. Because significant correlations of mGlu3 were seen with these three factors, and the schizophrenia and control groups differed significantly in mean age, pH and PMI (Table 2), we selected a sub-sample (‘matched sample’) which were matched on these variables. Diagnostic comparisons were made using this sub-sample by ANOVA, with any variable still showing a significant correlation included as a covariate (see Suckling, 2011). For further details, see Results.

## 3 Results

This study had two main components. First, we assessed several commercially available anti-mGlu3 antibodies, and one novel antibody directed at the mGlu3Δ4 variant, in knockout (versus wildtype) mouse brain, and in transfected (versus un-transfected) HEK293T/17 cells, for western blotting. Second, we tested the one well-validated antibody for its suitability for immunohistochemistry, and used it for a semi-quantitative immunoblotting study of mGlu3 in schizophrenia.

**Fig. 1:**
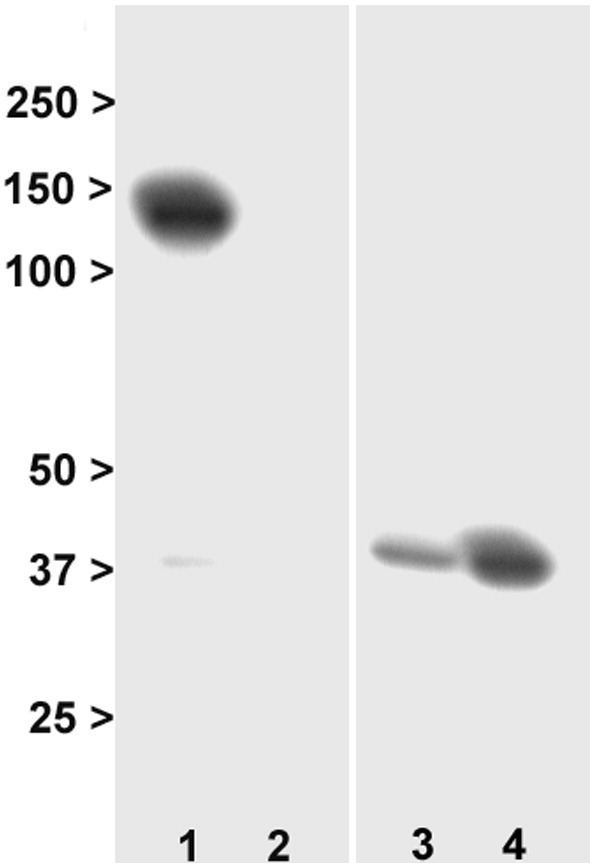
Characterisation of membrane and cytosolic fractions. Lane 1: membrane fraction, N-cadherin. Lane 2: cytosol fraction, N-cadherin. Lane 3: membrane fraction, GAPDH. Lane 4: cytosolic fraction, GAPDH. The blots show the predicted localisation of N-cadherin to the membrane, and the enrichment of GAPDH in cytosol. Numbers are molecular weight (kDa).

All western blot experiments, except where stated, were carried out on the membrane fraction. Fig. 1 demonstrates that the protocol resulted in the anticipated detection of the membrane protein N-cadherin in the membrane but not cytosolic fraction, and enrichment of GAPDH in the cytosolic fraction.

### 3.1 Initial characterisation of anti-mGlu3 antibodies

**Supplementary Fig. 1:**
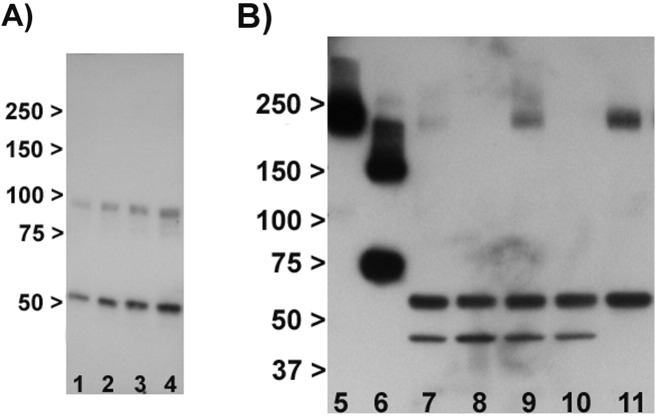
Examples of tested antibodies. A: Testing of sc-47138 in mouse brain. Lanes 1 and 3: wild-type. Lanes 2 and 4: Grm2^−/−^/3^−/−^, showing no difference between genotypes. B: Testing of N-terminal ab188750. 5: HEK293T/17 cells transfected with GRM3 cDNA. 6: HEK293T/17 cells transfected with GRM3Δ4 cDNA. 7: wild-type mouse brain. 8: Grm2^−/−^/3^−/−^ mouse brain. 9: wild-type mouse brain. 10: Grm3^−/−^ mouse brain. 11: human superior temporal cortex. Note bands at ~ 200 kDa in lanes 5,7,9 and 11 which are specific, as judged by their presence in transfected HEK293/17 cells (lane 5) and their absence in the two knockout mice (lanes 8 and 10). Note also the additional smaller, non-specific, bands present in all brain samples (lanes 7–11).

We first tested the anti-mGlu3 antibodies to see whether they produced bands of the predicted size for mGlu3 (~ 90–100 kDa and ~ 200 kDa) in mouse brain. Three antibodies (sc-47137, sc-47139 and ab10309) did not produce bands of these sizes but instead resulted in several smaller bands, and with a similar banding pattern in Grm2^−/−^/3^−/−^ as in wild-type mice (data not shown). One antibody (sc-47138) produced a band at ~ 90 kDa, as well as one at ~ 50 kDa; both bands were seen equally in Grm2^−/−^/3^−/−^ as well as wild-type mice (Supplementary Fig. 1A). Hence, none of these antibodies showed convincing evidence of binding to mGlu3 in our hands, and they were not studied further.

One antibody, ab188750, directed against the N-terminus, detected a strong band at ~ 200 kDa in wild-type mouse which was absent in Grm2^−/−^/3^−/−^ and Grm3^−/−^ mice (Supplementary Fig. 1B). Moreover, a band of the same size was also seen in cells over-expressing GRM3 and in human brain tissue. However, in addition, the antibody detected two smaller bands (of ~ 60 kDa and ~ 45 kDa) which were present in Grm2^−/−^/3^−/−^ and Grm3^−/−^ wild-type mice and hence non-specific; the 60 kDa band was also seen in human brain. This antibody also proved to give variable and sometimes weak staining (though with a similar pattern) across experiments.

**Fig. 2:**
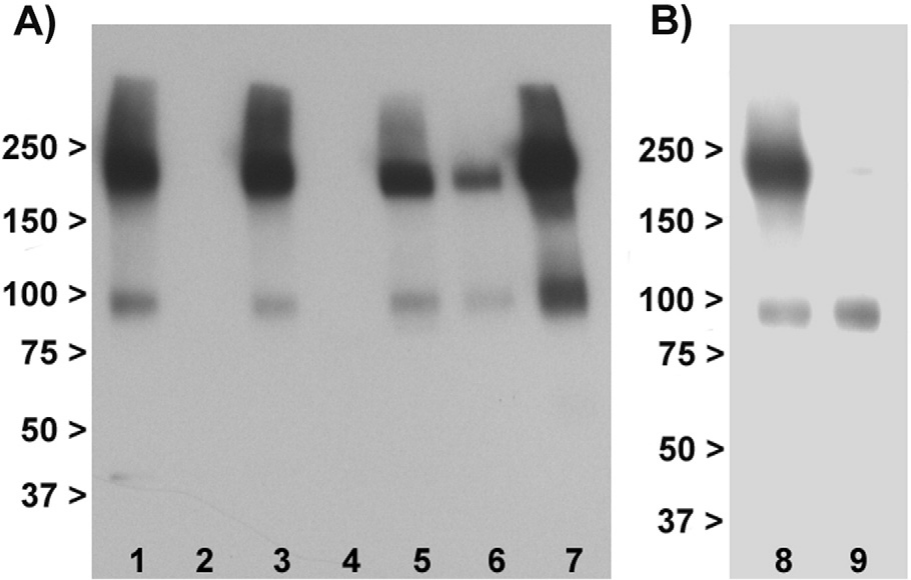
Western blotting with ab166608 antibody. Panel A: Immunoblotting profile in membranes. Lane 1: Wild-type mouse brain. 2: Grm2^−/−^/3^−/−^ double knockout mouse brain. 3: Wild-type mouse brain. 4: Grm3^−/−^ knockout mouse brain. 5: Rat brain. 6: Human superior temporal cortex. 7: Transfected HEK293T/17 cells. Lanes 1, 3, and 5–7 show the selective detection of two immunoreactive bands at the molecular weights predicted for monomeric and dimeric mGlu3 (~ 100 kDa and ~ 200 kDa respectively). The bands are specific to mGlu3 as judged by their absence in Grm2^−/−^/3^−/−^ and Grm3^−/−^ mice (lanes 2 and 4). Panel B: Staining with ab166608 antibody is concentrated in the membrane fraction (lane 8) relative to the cytosol (lane 9); note the latter also lacks a band corresponding to the dimer. Numbers are molecular weight (kDa).

The final antibody, ab166608, directed against the C terminus, proved most successful (Fig. 2A). It produced two strong bands of the predicted molecular weight for mGlu3 in mouse brain, and in transfected HEK293T/17 cells, which were absent in Grm2^−/−^/3^−/−^ and Grm3^−/−^ mouse brain and in un-transfected cells. No other bands were seen. It also produced the same banding pattern in human brain. Immunoreactivity was concentrated in membranes compared to the cytosol (Fig. 2B). We therefore focused on this antibody for all subsequent work.

### 3.2 Identification of mGlu3Δ4 and characterisation of an anti-mGlu3Δ4 antibody

**Fig. 3:**
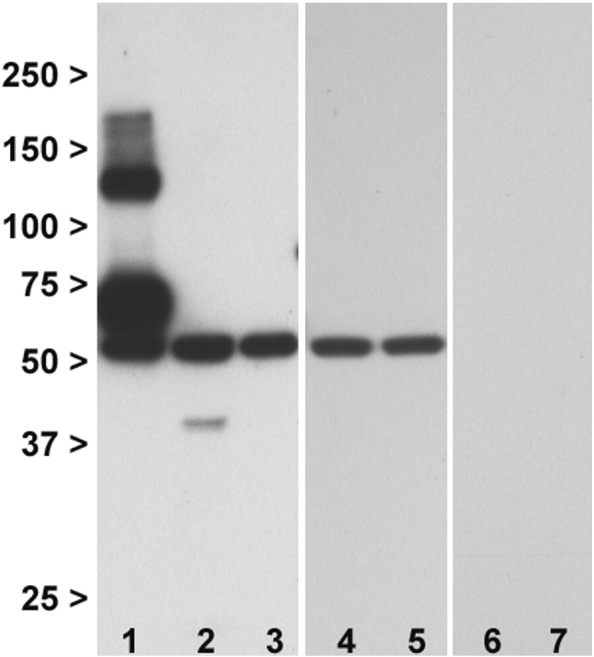
Western blotting with a novel mGlu3Δ4 antibody. 1: HEK293T/17 cells transfected with GRM3Δ4 cDNA. 2: Human superior temporal cortex. 3: Untransfected HEK293T/17 cells. 4: Wild-type mouse brain. 5: Grm2^−/−^/3^−/−^ mouse brain. 6: HEK293T/17 cells transfected with GRM3Δ4 mRNA in presence of blocking peptide. 7: Human superior temporal cortex, in presence of blocking peptide. The bands at ~ 65-70 kDa and ~ 140 kDa are specific, being seen in transfected cells (lane 1) but not in untransfected cells (lane 3) nor with the blocking peptide (lane 6). No bands of this weight are seen in human brain (lane 2). There is an additional band at ~ 60 kDa which is not detecting mGlu3Δ4 (as judged by its presence in lanes 3–5), despite being abolished by the blocking peptide (lanes 6 and 7). Numbers are molecular weight (kDa).

In a previous study (Sartorius et al., 2008), a GRM3 transcript isoform lacking exon 4 was identified. It was predicted to encode an mGlu3 variant with a novel C-terminus (due to a frameshift) of molecular weight of ~ 65 kDa, which we denote mGlu3Δ4. In this study, we transfected HEK293T/17 cells with GRM3Δ4, resulting in a band of this size for mGlu3Δ4 which was detected, as predicted, by the N-terminal ab188750 antibody (Supplementary Fig. 1B, lane 6) but not by the C-terminal ab166608 antibody (data not shown). We also tested a potential novel mGlu3Δ4-selective antibody (since the antibody used by Sartorius and colleagues was not available), and found that it also detected a band of approximately this size in transfected HEK293T/17 cells, as well as a larger band which may be a dimer (Fig. 3). These bands were specific, in that they were not seen with peptide block, or in un-transfected cells. No bands of similar sizes were detected in human brain, either in superior temporal cortex or in other regions tested (hippocampus, cerebellum, and cingulate cortex; data not shown), despite using a range of experimental conditions. Moreover, the antibody also detected a strong, smaller band (~ 60 kDa) which was present in un-transfected cells, human brain tissue, and brain tissue from wild-type mice (which do not express this variant) and Grm2^−/−^/3^−/−^ mice, and which is therefore non-specific (Fig. 3).

### 3.3 Immunocytochemistry and immunohistochemistry using the ab166608 antibody

**Supplementary Fig. 2:**
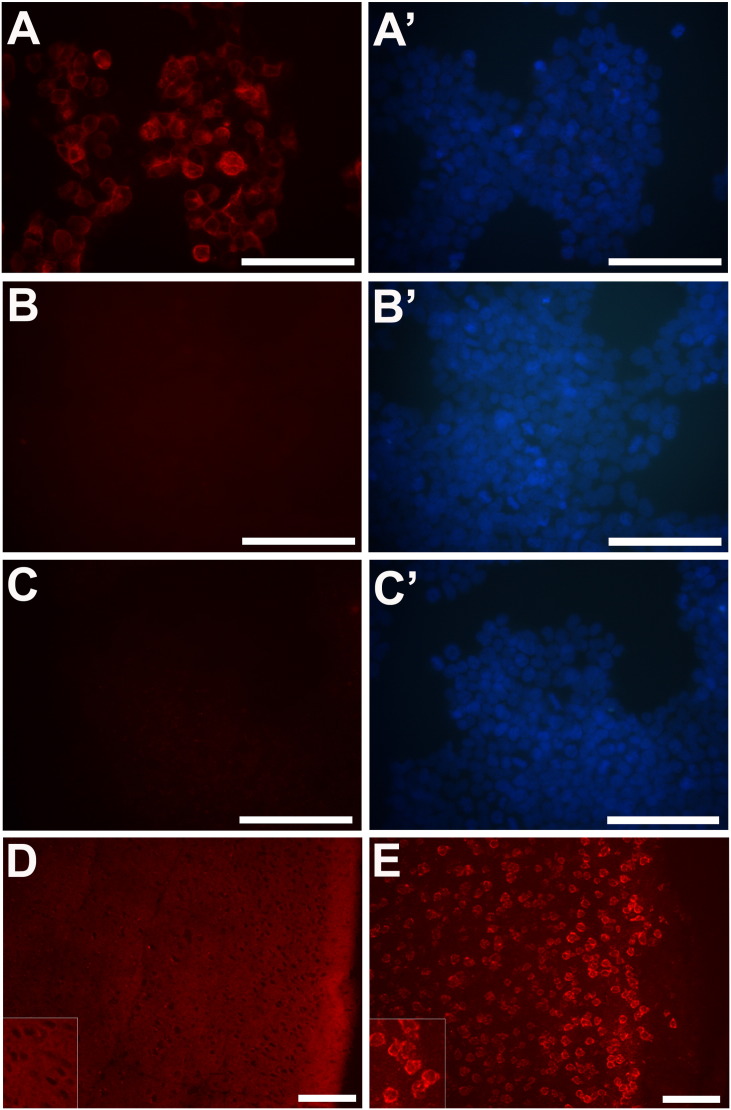
mGlu3 immunoreactivity in HEK293T/17 cells and mouse brain using the ab166608 antibody. A: Transfected HEK293T/17 cells, with DAPI stain shown in panel A′. B: Untransfected HEK293T/17 cells, with DAPI stain shown in panel B′. C: Transfected HEK293T/17 cells in absence of primary antibody, and DAPI stain shown in panel C′. D: wild-type mouse cortex, with pial surface to the right. E: Section from Grm2^−/−^/3^−/−^ mouse cortex. The insets in D and E show an enlarged magnification. Bar in each panel: 100 μm.

Since the cellular and subcellular localization of mGlu3 are of interest, we used the ab166608 antibody in immunocytochemistry of HEK293T/17 cells and immunohistochemistry in brain sections (Supplementary Fig. 2).

In the over-expressing HEK293T/17 cells, we found robust cytoplasmic mGlu3 immunoreactivity with enhanced signal around the plasma membrane. No immunoreactivity was seen in un-transfected cells, or in the absence of primary antibody. However, in wild-type mouse brain, diffuse neuropil immunostaining was seen, with sparing of neuronal cell bodies. The immunostaining was specific, as defined by the fact that it was abolished by the blocking peptide and not seen with omission of the primary antibody. A similar pattern of results was also seen in sections of human brain (data not shown). However, critically, in Grm2^−/−^/3^−/−^ mouse brain, a different pattern was observed, with strong immunoreactivity concentrated over cells, particularly around the plasma membrane, and with much less neuropil staining. This striking (but artefactual) staining was consistent in different wild-type and Grm2^−/−^/3^−/−^ mice.

### 3.4 mGlu3 immunoreactivity in human brain

We used the C-terminal antibody for quantitative assessment of mGlu3 immunoreactivity in human superior temporal cortex (Table 2) using western blots.

**Fig. 4:**
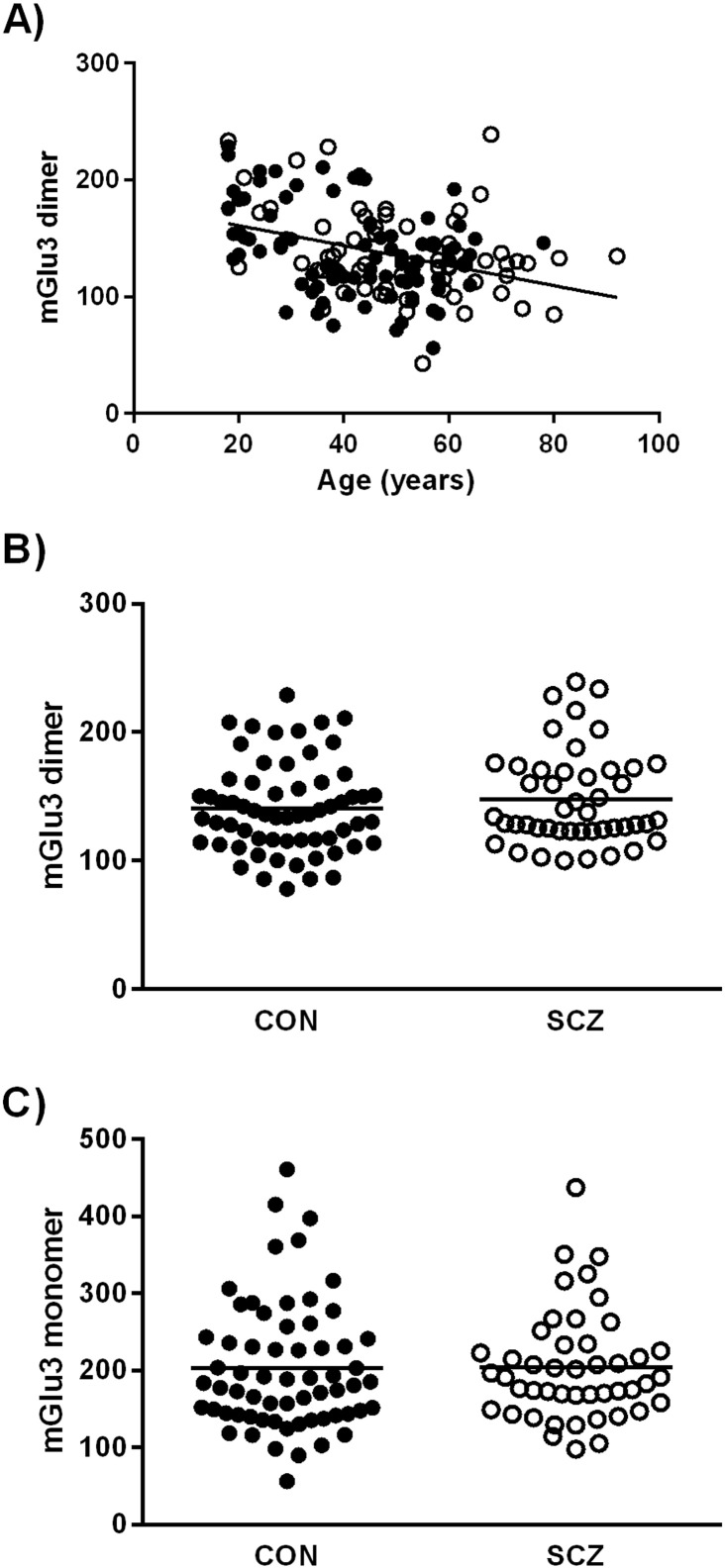
mGlu3 immunoreactivity in human superior temporal gyrus in relation to age and schizophrenia. A: Dimeric mGlu3 is inversely correlated with age. B: Dimeric mGlu3 in control subjects (filled circles) and subjects with schizophrenia (unfilled circles). C: Monomeric mGlu3 in control subjects (filled circles) and subjects with schizophrenia (unfilled circles). There are no differences between groups. Line in A shows correlation; lines in B and C show uncorrected means. In all panels, the y-axis units are mGlu3 immunoreactivity relative to the internal standards, which were set at 100.

mGlu3 immunoreactivity correlated negatively with age at death and PMI and positively with pH. This applied to dimeric and monomeric forms, and to control and schizophrenia groups considered separately (Table 4 and Fig. 4A). mGlu3 immunoreactivity was higher in men than women, both for the dimer (145 ± 38 vs. 127 ± 35; *t* = 2.94, d.f. 154, *p* = 0.004) and the monomer (205 ± 79 vs. 173 ± 68; *t* = 2.50, d.f. 154, *p* = 0.014); similar but non-significant differences were seen in the control and schizophrenia groups considered separately (data not shown). There were no effects of race, hemisphere, brain weight, smoking history, nor ethanol level, on mGlu3 immunoreactivity (data not shown).

### 3.5 mGlu3 immunoreactivity in schizophrenia

Because of the correlations of mGlu3 immunoreactivity with age, pH and PMI, and the fact that these variables differed significantly between controls and cases in the whole sample which would confound their use as covariates (Table 2), we performed our case-control comparisons on a sub-sample (of 63 controls and 46 patients with schizophrenia) which was matched on these variables (a ‘matched’ series; Table 2). Within this sub-sample, mGlu3 immunoreactivity still correlated negatively with age, and equivocally with PMI, but not with pH. Our primary group comparisons therefore included age and PMI as covariates.

**Fig. 5:**
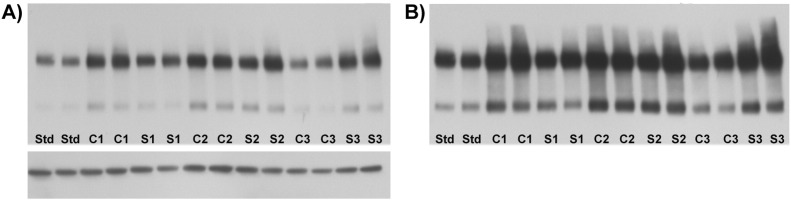
Representative western blots from the comparison of control subjects and subjects with schizophrenia. A: Example of a gel, containing duplicate standards (Std), three controls (C1–C3) and three schizophrenia subjects (S1–S3). This exposure was used for quantitation of the dimer (upper band). The bottom band is beta-actin. B: Same gel, at longer exposure, for quantitation of the lower and weaker monomer band.

Representative gels are shown in Fig. 5, and the results for mGlu3 immunoreactivity in schizophrenia compared to the control subjects are shown in Fig. 4. Neither mGlu3 dimer (Fig. 4B) nor monomer (Fig. 4C) differed between the groups (dimer: *F*_1, 105_ = 2.53, *p* = 0.115; monomer: *F*_1, 105_ = 0.43, *p* = 0.51). Nor was a diagnostic effect observed for the dimer: monomer ratio (*F*_1, 105_ = 0.09, *p* = 0.77) or for the sum of dimer and monomer bands (data not shown). Beta-actin, used as a loading control, did not differ between groups, and a negative result was also seen if mGlu3 was normalised to beta-actin rather than to the internal pooled standard (data not shown). Note in Fig. 4 that the y axis units are relative to the internal standard, which was set to 100 (see Section 2.8). It is thus apparent that both case and control groups had mean band densities greater than the mean of the six subjects who were used to generate the internal standard. However, this issue did not lead to film saturation or a ‘ceiling effect’, since longer exposures led to darker bands (compare Fig. 5A and 5B). Moreover, had saturation occurred, we would not have observed the correlations of mGlu3 with age, post mortem interval and pH (Table 4). The disparity in band intensities between the internal standard pool and the experimental samples likely arose for two reasons. First, compared to the brain series as a whole, the six subjects used for the internal standards had, by chance, a greater age, longer post mortem interval, and lower pH (Supplementary Table 1 and Table 2); all of these factors are associated with reduced mGlu3 immunoreactivity (Table 4). Second, the samples used for the internal standards had undergone three freeze-thaw cycles, whereas the study samples had only one. Given the equivocal sex dimorphism in mGlu3 immunoreactivity noted above, we repeated the ANOVAs with sex as an additional factor; no main effect of sex, nor a diagnosis-by-sex interaction, was seen for either dimer or monomer (data not shown). However, in a post hoc analysis, mGlu3 dimer was increased in men with schizophrenia compared to male controls (156 ± 38 vs. 143 ± 35; *F*_1, 74_ = 3.91, *p* = 0.050), with no difference for women (134 ± 36 vs. 129 ± 26; *F*_1, 27_ = 0.32, *p* = 0.57).

Within the full schizophrenia sample, monomeric mGlu3 was negatively correlated with age at onset of illness (*N* = 70; *R* = − 0.321, *p* < 0.01); however this correlation was weaker and non-significant in the sub-sample used for case-control comparisons (*N* = 46; *R* = − 0.171, *p* = 0.25). Monomeric and dimeric mGlu3 correlated negatively and significantly with duration of illness (in the full sample, and in the matched sample), but these correlations disappeared when partialling for age at death (data not shown). mGlu3 immunoreactivity did not correlate with any indices of antipsychotic exposure (last recorded dose; cumulative exposure), nor did it differ between those with positive and negative toxicology, nor was it influenced by whether the patient had died by suicide or natural causes (data not shown).

### 3.6 mGlu3 and GRM3 genotype

We assessed whether rs10234440 genotype predicted mGlu3 immunoreactivity. Because of the low frequency of the rare (C) allele, we compared TT homozygotes with C carriers for this analysis. We found no effect of genotype on monomeric or dimeric mGlu3 (Table 5). Neither did we find any genotype-by-diagnosis interactions (data not shown).

## 4 Discussion

### 4.1 Characterisation of antibodies

It is increasingly recognised that many antibodies used as experimental tools have not been sufficiently well characterised, and there are many examples of false positive and unrepeatable findings, as well as the expenditure of time and money on antibodies which fail to perform as the vendor claimed (Saper and Sawchenko, 2003, Rhodes and Trimmer, 2006). These issues affect receptors of interest in schizophrenia research, including mGlu1 receptors (Ayala et al., 2012) and muscarinic receptors (Pradidarcheep et al., 2008) just as much as other target proteins. Reflecting these concerns, there are calls for more careful, multifaceted, and method-specific validation of antibodies (Holmseth et al., 2006, Lorincz and Nusser, 2008, Bordeaux et al., 2010, Baker, 2015). Amongst other considerations, appropriate characterisation should include demonstration that the antibody binds specifically and selectively to band(s) of the predicted molecular weight of the target protein when present, and with absence of signal when the target protein is absent. These criteria are most readily assessed by showing that immunoreactivity is present in a wild-type mouse but not in a mouse with the encoding gene knocked out (constitutively or conditionally), and by showing that the bands are seen when cells are transfected with the encoding gene and thence express the protein.

To our knowledge, no commercially available anti-mGlu3 antibodies have previously been characterised using either approach; the one exception in schizophrenia is provided by Corti et al. (2007) but their anti-mGlu3 antibody was generated in-house and not available to others. Our present results confirm that, in our hands and for the batch(es) and experimental conditions used, several anti-mGlu3 antibodies fail, as judged by bands of the wrong size and/or bands which are not abolished in Grm3^−/−^ or Grm2^−/−^/3^−/−^ mice. We also had similar experiences with other anti-mGlu3 and anti-mGlu2/3 antibodies tried previously (TAL and PJH, unpublished observations). One antibody tested here, ab188750, did successfully detect a specific band at ~ 200 kDa, the putative mGlu3 dimer, and therefore could be used for immunoblotting for that target. However, it did not detect the mGlu3 monomer, and also detected non-specific smaller bands, limiting its utility.

Hence, of the six anti-mGlu3 antibodies tested, only one (ab166608) met our criteria in knockout mice, and in transfected cells, and in human brain. Using this antibody, we made several observations. Firstly, we confirm the presence of two bands for mGlu3; one being the predicted molecular weight of the full length protein (~ 95 kDa); we (and others; Corti et al., 2007) assume the second, and more intense, band at ~ 200 kDa is a homodimer, as occurs for many other GPCRs, and with the dimer being the primary functional unit (El Moustaine et al., 2012). It is interesting that the N-terminal antibody (ab188750) only detected the dimer (in membranes); we have no explanation but speculate that this reflects a conformational change such that the epitope is only exposed when in dimeric form.

Despite its validation for western blots, we did not find ab166608 suitable for immunohistochemistry in brain tissue because of the signal seen in Grm2^−/−^/3^−/−^ mice. Notably, this latter signal appeared plausible, with enhanced staining around the cell membrane, consistent with the membrane enrichment seen in the western blots. These observations highlight the need for caution and for additional immunohistochemical controls even for antibodies validated by immunoblotting (Holmseth et al., 2006, Rhodes and Trimmer, 2006, Baker, 2015). We did not pursue more detailed strategies, such as pepsinisation, or antigen retrieval, which might have permitted different and more valid immunohistochemical findings, as was reported for anti-mGlu2 antibodies (Lorincz and Nusser, 2008).

### 4.2 Factors affecting mGlu3 immunoreactivity in human brain

A decline with age has been reported for frontal cortex mGlu3 immunoreactivity (Corti et al., 2007) and for GRM3 mRNA (Colantuoni et al., 2008). Age-related reductions are also seen in this region for mGlu2/3 binding site density (Frank et al., 2011) and for mGlu2/3 immunoreactivity in controls but not in schizophrenia subjects (Crook et al., 2002). Our findings (Table 4 and Fig 4A) confirm that there is a robust decrease of mGlu3 across adulthood in human brain, occurring in superior temporal cortex as well as prefrontal cortex. It is unknown whether the reduction occurs equally in all cells or compartments expressing mGlu3, or occurs preferentially in one or other location (e.g. in glia, or presynaptically). Our failure to identify an anti-mGlu3 antibody suitable for immunohistochemistry in human brain complicates investigation of this issue.

Correlations between gene expression and pH, and to a lesser extent PMI, are well recognised for mRNAs. The present data emphasise that, despite generally greater stability of proteins to such factors (Harrison et al., 1995, Stan et al., 2006), similar concerns can apply to protein products (Wang et al., 2000, Eastwood and Harrison, 2001, Ferrer et al., 2007), in this case mGlu3 (Table 4). Thus demographic and perimortem factors require careful attention in quantitative western blot (or immunohistochemical) studies, with inspection for the presence of correlations, matching for variables between groups, and appropriate statistical control for their influence. In the current study, these issues mandated the selection of a subgroup of the whole sample. Fortunately, the initial sample size meant that the subgroup was still substantial, and considerably larger than all prior studies of mGlu3 in schizophrenia.

### 4.3 mGlu3 immunoreactivity in relation to schizophrenia and GRM3 genotype

Previous studies of mGlu3 immunoreactivity in schizophrenia had not produced a consistent pattern of results (Table 1), and studies of GRM3 mRNA had similarly not revealed clear evidence of altered expression (Harrison et al., 2008, Sartorius et al., 2008). The mGlu3 studies were limited by the fact that two studies had used antibodies which cross-reacted with mGlu2 (Crook et al., 2002, Gupta et al., 2005), and one used an anti-mGlu3 antibody with only limited characterisation (Ghose et al., 2009), and all three were relatively limited in sample size. The study by Corti et al. (2007) had the merit of a larger sample (35 controls, 20 cases), and the use of a novel anti-mGlu3 antibody which had been validated in Grm3^−/−^ mouse brain. Their main finding in schizophrenia was a reduction in the putative dimeric form of mGlu3 in prefrontal cortex, without any alteration in the monomer. However, we did not replicate this result in our sample (63 controls, 46 cases), finding no change in either mGlu3 immunoreactive band. Clearly, we cannot extrapolate our results beyond the superior temporal cortex, but overall the data support the conclusion that there are no alterations in mGlu3 expression in schizophrenia. Our data did suggest the possibility of an increase in mGlu3 dimer immunoreactivity in men with schizophrenia, but we do not view this as robust in the absence of a diagnosis-by-sex interaction, or any prior reason for predicting such a result. The absence of any correlations between mGlu3 and medication exposure, or smoking history, suggests that the lack of any diagnostic difference in mGlu3 immunoreactivity does not arise from antipsychotic- or smoking-induced normalisation of an underlying change in mGlu3 immunoreactivity.

Our study used total membrane protein extracted from homogenates of the superior temporal cortex of patients with chronic schizophrenia. It remains possible that in schizophrenia there are localised alterations (e.g. in particular cell populations, or in other brain regions), or changes which occurred prior to the onset of illness, or which are limited to particular phases of the illness (c.f. the possibility that mGlu2/3 agonists are effective early but not late in the disease; Kinon et al., 2015, Krystal and Anticevic, 2015). Another possibility not addressed by the current methodology is that the subcellular distribution of mGlu3 is altered in schizophrenia; for example, there could be a shift between pre- and post-synaptic domains, or between intracellular and plasma membranes. Indeed, the latter has been reported for ionotropic glutamate receptors and other proteins, indicative of aberrant trafficking (Hammond et al., 2010, Hammond et al., 2012, Kristiansen et al., 2010). Investigating whether this also occurs for mGlu3 would be of interest, but would require much more complex fractionation procedures than used here (Behan et al., 2009, Hahn et al., 2009, McCullumsmith and Meador-Woodruff, 2011), and would be challenging to implement in such a large series of brains. A complementary approach to study the intracellular distribution of mGlu3 would be to use immunohistochemistry, but the unsuitability of ab166608 precluded this method. Finally, there could also be functional effects on mGlu3 signalling in schizophrenia which are independent of receptor abundance, for example differences in heterodimerisation with other receptors (Gonzalez-Maeso et al., 2008, Doumazane et al., 2011), or the coupling of mGlu3 to G-proteins and other downstream effectors (Salah-Uddin et al., 2009).

The GRM3 polymorphism we studied did not influence mGlu3 immunoreactivity. The SNP, rs10234440, is within the GRM3 locus which shows genome-wide association to schizophrenia (Schizophrenia Working Group of the Psychiatric Genomics Consortium, 2014). The negative result therefore provides no evidence that the risk locus operates by modulating GRM3 expression, and alternative explanations should be sought. However, there are many caveats to this conclusion. For example, the risk variant is intronic and hence may alter splicing rather than transcription (as suggested by the earlier study which found an association between a GRM3 risk SNP and the GRM3Δ4 mRNA isoform; Sartorius et al., 2008). However, we were unable to investigate this possibility in terms of the abundance of the encoded mGlu3Δ4 variant, because the new antibody generated against this sequence proved unsuccessful, failing to detect the variant in human brain tissue but instead detecting a smaller and non-specific band. Other possible mechanisms of genetic association include modulation of an antisense GRM3 transcript (Buonaguro et al., 2015), or effects of genotype on mGlu3 which are limited to specific locations or developmental stages, as is the case for some other schizophrenia risk genes (Kleinman et al., 2011, Birnbaum et al., 2014, Paterson et al., 2014, Tao et al., 2014).

## 5 Conclusions

We draw two main conclusions from this work. The first is that many antibodies available to study mGlu3 are non-specific as judged using knockout mice and other experimental controls. Hence the value of such mice – as well as cell transfection and overexpression – as part of a comprehensive antibody characterisation is highlighted. Without this information, there is a significant potential for misleading findings using immunoblotting (and immunohistochemistry). The second conclusion is that the abundance of membrane-bound mGlu3 is not altered in the superior temporal cortex in schizophrenia. This is a robust conclusion, given the large sample size, and the use of a well-validated, specific antibody. However, it leaves open the possibility of more nuanced alterations in mGlu3 expression, and of aberrant receptor signalling, in the disorder. These possibilities are worth pursuing, given the evidence that GRM3 contributes to the genetic risk for schizophrenia, and in light of the continuing focus on group II metabotropic glutamate receptors as therapeutic targets.

The following are the supplementary data related to this article.

Supplementary Table 1 Subjects pooled for internal protein control sample. Supplementary Table 1

## Role of funding source

AGB is funded by a Fellowship from the Fundación Alfonso Martín Escudero. Work supported by a Wellcome Trust Strategic Award (102616/Z) and the United Kingdom Medical Research Council (G0801747).

## Conflict of interest

PJH has served as an expert witness on patent litigation regarding drugs in current use to treat schizophrenia. The other authors report no conflicts of interest.

## Contributors

PJH, TAL and AG designed the study. PJH, TAL and AG drafted the manuscript. AG, TAL and MAW carried out the experiments. TMH and JEK provided the post mortem tissue. TMH and JEK critically reviewed the manuscript. All authors contributed to and have approved the final manuscript.

## Figures and Tables

**Table 1:** Prior western blot studies of mGlu3 in schizophrenia, showing main methodological features and key findings.

Study	Antibody	Antibody validation	Sample	Sample preparation and method^a^	Brain area^b^	Loading controls	Bands measured	Main findings
Crook et al. (2002)	Anti-mGlu2/3 (Chemicon)	Pre-absorption with mGlu2/3 peptide abolished immunoreactivity.	20 SCZ, 20 CON	Membranes; 7.5% PA gels and 50 μg protein (duplicates).	BA46	Two internal controls in each blot.	~ 100 kDa	No group difference. Negative correlation with age in controls.
Gupta et al. (2005)	Anti-mGlu2/3 (Upstate Bio-technology)	No data presented; paper refers to earlier papers, but the latter used a Chemicon mGlu2/3 antibody (with peptide pre-absorption used as the control).	16 SCZ, 9 CON	Total homogenates; 5% β-ME and heated at 95 °C for 4 min; 7.5% PA gels and 40 μg protein (duplicates).	BA9, 11, 32 and 46; n. accumbens, putamen, caudate	None stated.	~ 100–110 kDa	Increase in BA46 in schizophrenia.
Corti et al. (2007)	Anti-mGlu3, raised against residues 16–35 in mouse mGlu3.	Tested using GRM3 cDNA-transfected CHO cells, and grm3^−/−^ mouse brain.	20 SCZ, 35 CON	Membranes; 20 mM DTT, heated at 60 °C for 3 min; 8% PA gels 40 μg protein (triplicates).	BA10	β-actin and Ponceau S. Each gel also contained two internal standards.	~ 100 and ~ 200 kDa bands in cells and WT mouse brain; not seen in grm3^−/−^ mouse. Human brain: bands at ~ 200 kDa and doublet at ~ 95/100 kDa.	200 kDa band reduced in schizophrenia. Negative correlations with age for 200 kDa and 100 kDa bands.
Ghose et al. (2009)	Anti-mGlu3 (Abcam)	No data presented; the authors refer to a prior paper, but the latter does not provide clarity.	15 SCZ, 15 CON	Total homogenates; 10% PA gels and 20 μg protein (duplicates).	Prefrontal, temporal and motor cortex.	β-tubulin	Monomer; Molecular weight not specified.	mGlu3 decreased in prefrontal cortex in schizophrenia.

a DTT: dithiothreitol. ME: mercaptoethanol. PA: polyacrylamide.

b BA: Brodmann area. BA9/46: dorsolateral prefrontal cortex. BA10: frontal pole. BA11, BA32: medial prefrontal cortex.

**Table 2:** Demographic details of subjects studied.

	Full series		‘Matched’ series^a^	
	Controls	Schizophrenia	Controls	Schizophrenia
Number	87	70	63	46
Sex (male/female)	59/28	43/27	46/17	32/14
Age (years)	42.0 (14.7)	51.6 (15.5)⁎	42.7 (14.3)	46.7 (13.9)
Race (C/AA/other)^b^	28/52/7	26/40/4	21/38/4	18/25/3
Hemisphere (R/L/not known)^c^	15/59/13	9/55/6	9/46/8	7/36/3
Brain weight (grams)	1372 (152)	1327 (161)	1377 (151)	1351 (153)
Brain pH	6.55 (0.33)	6.37 (0.29)⁎	6.56 (0.21)	6.47 (0.23)
Post mortem interval (hours)	32.5 (14.5)	40.1 (19.8)⁎	33.5 (14.4)	34.6 (13.9)
Smoker (yes/no/not known)	21/63/3	51/19/0	16/44/3	37/9/0
Suicide (yes/no/not known)	0/87/0	11/58/1	0/63/0	10/36/0
Age at onset of illness (years)	–	23.3 (9.1)	–	20.5 (6.0)
Duration of illness (years)	–	28.3 (14.2)	–	26.3 (12.7)
Antipsychotic detected (yes/no)	–	42/28	–	27/19
Daily CPZ equivalents (grams)^d^	–	600 (100 − 3000)	–	600 (100–3000)
rs10234440 genotype (TT/TC/CC)^e^	65/19/2	56/12/2	46/15/1	38/7/1

Values are mean (SD).

⁎ Cases vs. controls, *p* < 0.01 (*t*-test).

a Limited to subjects aged < 72 years, with post mortem interval < 72 h, and brain pH between 6.1 and 6.9. Cases and controls in this ‘matched’ series do not differ in age, pH or PMI (all *p* > 0.05).

b C: Caucasian. AA: African American.

c R: right. L: left.

d CPZ: chlorpromazine. Range in brackets.

e Not available for one control subject.

**Table 3:** Antibody details.

Antibody	Supplier	Product code	Batch	mGlu3 epitope	Concentration used		
					WB	ICC	ICH
mGlu3	Santa Cruz	sc-47137	A0907	N-terminal domain	1:200	–	–
mGlu3	Santa Cruz	sc-47138	D1913	N-terminal domain	1:200	–	–
mGlu3	Santa Cruz	sc-47139	B2614	N-terminal domain	1:200	–	–
mGlu3	Abcam	ab10309	GR11670-6	Residues 118–126	1:500	–	–
mGlu3	Abcam	ab188750	GR230043-1 GR230043-2	N-terminal domain	1:1000	–	–
mGlu3	Abcam	ab166608	YJ100911CS	845-C terminus	1:50,000	1:5000	1:10,000
mGlu3 peptide	Abcam	ab208352	GR250252-1	Peptide for ab166608	–	–	10 μg/mL
mGlu3Δ4	Cambridge Biomedicals	Custom made		TQGSHHPVSTPEEC	1:500	–	–
mGlu3Δ4 peptide	Cambridge Biomedicals	Custom made		TQGSHHPVSTPEEC	30 μg/mL	–	–
Βeta-actin	Sigma	A1978	011M4812	–	1:2,000,000	–	–
N-Cadherin	BD Biosciences	610920	78545	–	1:10,000	–	–
GAPDH	Abcam	ab9484	GR165366–3	–	1:5000	–	–
HRP goat anti-rabbit	Biorad	172-1019	350003011	–	1:10,000	–	–
HRP goat anti-mouse	Biorad	172-1011	350003068	–	1:5000	–	–
Alexa 568 goat anti- rabbit	Invitrogen	A11011	623962	–	–	1:1000	1:1000

**Table 4:** Correlations of mGlu3 immunoreactivity with age, pH and post mortem interval (PMI).

	Whole sample (*N* = 157)		Controls (*N* = 87)		Schizophrenia (*N* = 70)	
	Dimer	Monomer	Dimer	Monomer	Dimer	Monomer
Age (y)	− 0.360⁎⁎⁎	− 0.368⁎⁎⁎	− 0.411⁎⁎⁎	− 0.341⁎⁎	− 0.321⁎⁎	− 0.414⁎⁎⁎
pH	+ 0.328⁎⁎⁎	+ 0.232⁎⁎	+ 0.412⁎⁎⁎	+ 0.264⁎⁎	+ 0.232	+ 0.167
PMI (h)	− 0.249⁎⁎	− 0.179⁎	− 0.270⁎	− 0.153	− 0.236⁎	− 0.200

Values are Pearson coefficients.

⁎ *P* < 0.05.

⁎⁎ *P* < 0.01.

⁎⁎⁎ *P* < 0.001.

**Table 5:** mGlu3 immunoreactivity and rs10234440 genotype.

	TT homozygotes	C carriers
Whole sample	N = 120	N = 35
Monomer	196 (81)	185 (66)
Dimer	141 (39)	132 (30)
Controls	N = 65	N = 21
Monomer	202 (85)	182 (67)
Dimer	143 (40)	128 (31)
Schizophrenia	N = 55	N = 14
Monomer	188 (74)	190(66)
Dimer	137 (39)	139 (29)

Values are mean (SD) normalised to internal standard (arbitrary units). There are no differences between groups.
