# Supplementary material for: Metabotropic glutamate receptor 3 (mGlu3; mGluR3; GRM3) in schizophrenia: Antibody characterisation and a semi-quantitative western blot study
Source: Schizophr Res. 2016 Nov;177(1-3):18–27. doi: 10.1016/j.schres.2016.04.015 (PMC5145804; doi:10.1016/j.schres.2016.04.015)
Supplement: Supplementary Table 1 — Subjects pooled for internal protein control sample. [file mmc1.docx]

**Supplementary table 1: Subjects pooled for internal protein control sample.**

| **Diagnosis** | **Sex** | **Age** | **PMI (h)** | **pH** |
| --- | --- | --- | --- | --- |
| SCZ | F | 36 | 20 | 5.8 |
| CON | F | 50 | 51 | 6.0 |
| CON | M | 44 | 29 | 6.8 |
| SCZ | M | 52 | 60 | 6.0 |
| SCZ | M | 61 | 34 | 6.1 |
| CON | F | 65 | 58 | 6.6 |
|  |  |  |  |  |
| **Mean** | - | 51 | 42 | 6.2 |

Scz: schizophrenia. Con: control.
